# The Case for Whole-Person Integrative Care

**DOI:** 10.3390/medicina57070677

**Published:** 2021-06-30

**Authors:** Wayne B. Jonas, Elena Rosenbaum

**Affiliations:** 1 Samueli Foundation, Alexandria, VA 22314, USA; 2 Department of Family and Community Medicine, Albany Medical College, Albany, NY 12208, USA; erosenbaum@communitycare.com

**Keywords:** integrative health, whole-person health, systems research, reductionism, complementary and alternative medicine, healing, healthcare transformation, overview

## Abstract

*Rationale:* There is a need for medicine to deliver more whole-person care. This is a narrative review of several models of whole-person care and studies that illustrate the business case for whole-person models in primary care. *Objectives:* To provide an overview of what whole-person care models exist and explore evidence to support these models. *Study Selection:* Representative whole-person care models widely used in the United States are summarized and evaluated. Selected studies focused on outpatient primary care with examples from programs that integrate the delivery of conventional medical care, complementary and alternative medicine, and self-care within the context of social and cultural environments. *Methods:* Pubmed search conducted December 2020–February 2021. Two iterative searches using terms for “Whole Health Veterans Administration”, “integrative medicine”, “integrative health”, “complementary and alternative medicine”, and, as they related to the outcomes, of “health outcomes”, “cost-effectiveness”, “cost reduction”, “patient satisfaction”, and “physician satisfaction”. Additional studies were identified from an initial search and the authors’ experience of over 50 years. We looked for studies of whole-person care used in general primary care, those not using a single modality and only from United States practices. *Results:* A total of 125 (out of 1746) studies were found and met our inclusion criteria. We found that whole-person models of primary care exist, are quite heterogeneous in their approaches, and routinely report substantial benefits for improving the patient experience, clinical outcomes and in reducing costs. *Conclusions:* Evidence for the benefit of whole-person care models exist but definitions are quite heterogenous and unfocused. There is a need for more standardization of whole-person models and more research using whole systems approaches rather than reductionistic attempts using isolated components.

## 1. Introduction

For the last 100 years, healthcare has been dominated by the relentless application of a certain strategy in science known as reductionism. In this strategy the person is divided into ever smaller parts including organs, tissues, cells, organelles, proteins, and finally genetic material—RNA and DNA. This research strategy involves looking at how these parts influence each other and designing ways to control those parts. From this we have an explosion in knowledge of the parts of an individual and their mostly physical component interactions. This “knowledge of the parts” is then applied to a whole person by attempting to control these physical processes when they seem to be involved in disease. Treatment is framed almost entirely around controlling these component parts and attempting to balance the effectiveness of this control against the interference this control also places on normal, non-disease processes, producing unwanted effects.

Our theory is that in pursing the reductionistic strategy in science, we have neglected development of more “holistic” approaches of evaluation that provide more direct and relevant value for what matters in health care. Almost inevitably, the attempt to control one part of a complex system like a whole human being results in only partial effects (a small degree of efficacy) and adverse (unwanted) effects. Frequently, this produces more problems than it solves, and is fundamentally not designed to enhance the salutogenic and adaptogenic processes that rebalance a person’s health as a whole [1,2]. In other words, the application of reductionism results in a focused attempt to control or remove the disease—to cure—rather than to restore the organism to optimal functioning—to heal.

Thus, there is a need to develop and use more “whole person” and “whole systems” strategies and evaluate their impact on health care outcomes, patient and provider experience and costs. This gap is now the focus of the strategic plan for the National Center for Complementary and Integrative Health (NCCIH), of the United States National Institutes of Health. [3] In his article we review some of these “whole person approaches” and evaluate their impact on the main outcomes desired in health care—clinical outcomes, experiences of care, and costs.

## 2. A Whole Person

A person is more than just their physical components or parts. They consist of the body and the external environment for sure but are also made up of at least three other dimensions including: (1) their behavior and lifestyle that interacts with the environment; (2) their social and emotional dimensions; and (3) their mental and spiritual dimensions, which bring meaning and purpose to life. These dimensions of what make up a human being have been defined by classic writers such as Abraham Maslow in the Hierarchy of Human Needs [4], Victor Frankel who demonstrated the importance of meaning for survival [5], and George Engel in his landmark work on the biopsychosocial model in biology and medicine [6]. 

How then can we develop health care that addresses the whole person in all his, her or their dimensions? Additionally, what evidence do we have that heath care models that incorporate whole person, rather than reductionistic models produce benefit on outcomes that matter, such as health outcomes, patient and provider experience and costs. In this narrative review, we describe several models of care that start from a holistic rather than a reductionistic perspective. We then review the selected literature for the impact these holistic approaches can have had on relevant outcomes. Our goal is to better, describe, define, and demonstrate the value of a new type of health care focused on the whole person. 

## 3. Examples of Whole-Person Models

### 3.1. Optimal Healing Environments

**Figure 1:**
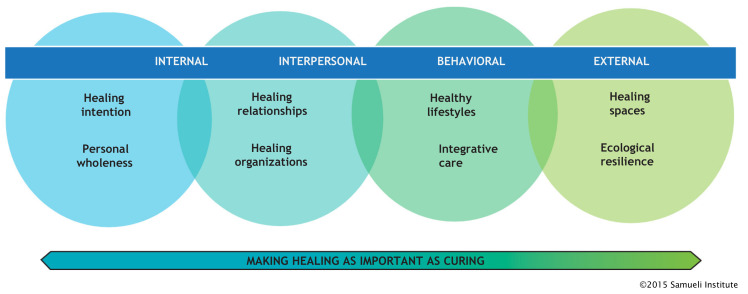
The Optimal Healing Environments Model.

Multiple models and operational frameworks have been created to conceptualize and deliver whole-person care. An example is the Optimal Healing Environment (OHE) framework developed by the Samueli Institute in 2003 [7]. Figure 1 illustrates this model. Several health care systems have used this framework for their operations and reported improved patient experience [8]. In addition, a comprehensive review of the business case benefits of this approach is published [9].

### 3.2. Total Force Fitness

**Figure 2:**
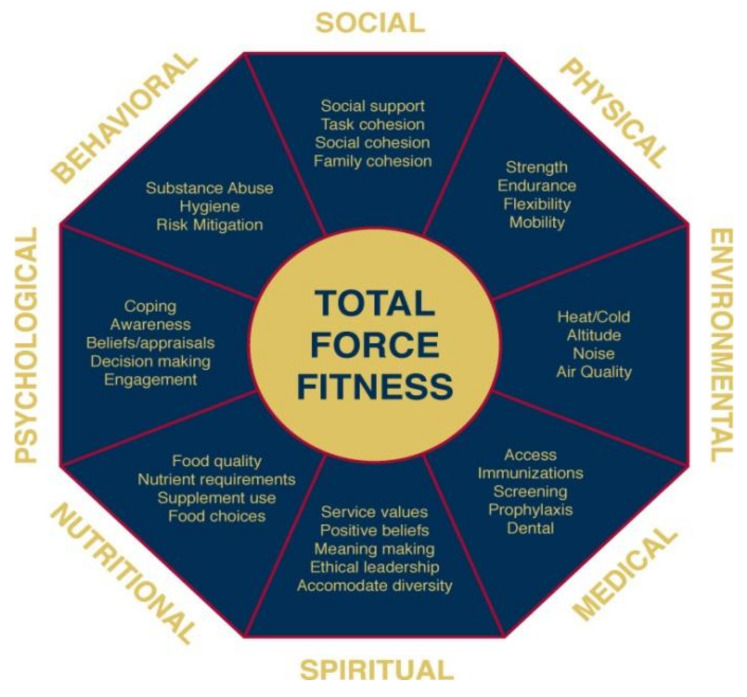
The Total Force Fitness Model.

Another example is the Total Force Fitness (TFF) model facilitated by the Samueli Institute and used by the US Military [10]. Figure 2 shows this model. TFF went beyond a medical environment to include physical, psychological, spiritual, social, and even economic aspects of human flourishing, integrated into a single framework and data collection system. This model sought to use a comprehensive whole-person framework for use in health care, as well as prevention and readiness within a military context. Multiple offshoots of this framework documented its value and are being applied in clinical settings such as the “Performance Triad” [11], in entire communities such as “Operation LiveWell”, and the latest and most sophisticated version applied to the entire enterprise called the Holistic Health and Fitness (H2F) Program [12]. This program “recognizes that readiness depends on the proper combinations of physical fitness (such as strength, speed, and endurance) and foundational health (such as the cardiovascular, respiratory, immune, and hormonal systems), which are optimized through careful attention to nutritional readiness, mental readiness, spiritual readiness, and sleep readiness”. HF2 aims to track the total health and function of a person in real-time to optimize function, not just treat disease. 

### 3.3. Whole Health

**Figure 3:**
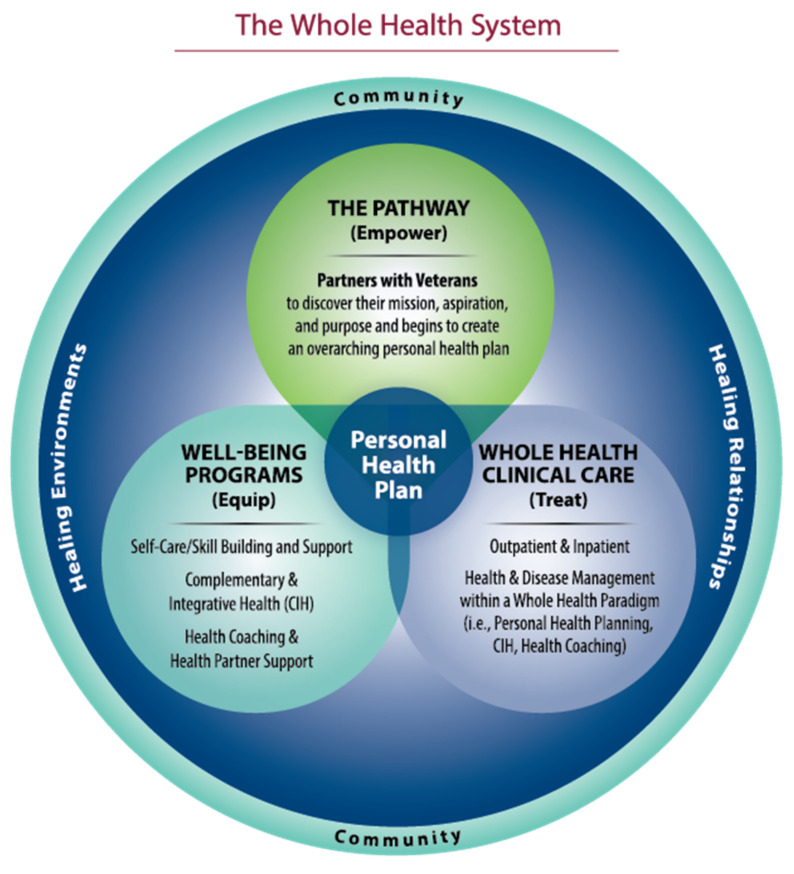
The VHA Whole Health Model.

Another, more recent model is currently being implemented in the Veteran’s Health Administration (VHA) called Whole Health [13]. This approach starts with the mental and spiritual dimension, asking patients “what matters” to them in their life—their meaning and purpose—and then capturing 10 areas of behavior, lifestyle, and social support to create a “personal health plan” based on those assessments. Recent research on the outcomes from this model has found significant improvements in all major areas and will be reported on later in this review. Figure 3 illustrates this model. 

### 3.4. Integrative Health

**Figure 4:**
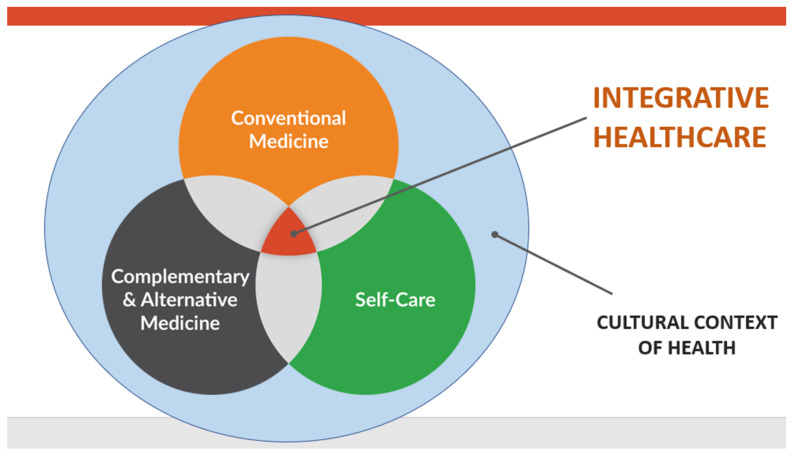
The Integrative Health Model.

The latest evolution of what has been called complementary and alternative medicine is integrative health. Integrative health involves the optimal combination of all evidence-based approaches to help heal the person as a whole. Integrative care defines itself as the appropriate merger and integration of conventional care, drugless approaches, including complementary and alternative medicine (CAM), and behavior and lifestyle medicine. Integrative medicine and integrative health are being used by over 70 academic health centers in the U.S. and Canada, which have formed an organization called the Academic Consortium for Integrative Medicine and Health. The University of California, Irvine is the first major University to completely embrace this approach with others following [14,15]. Figure 4 illustrates this approach.

### 3.5. Advanced Primacy Care

**Figure 5:**
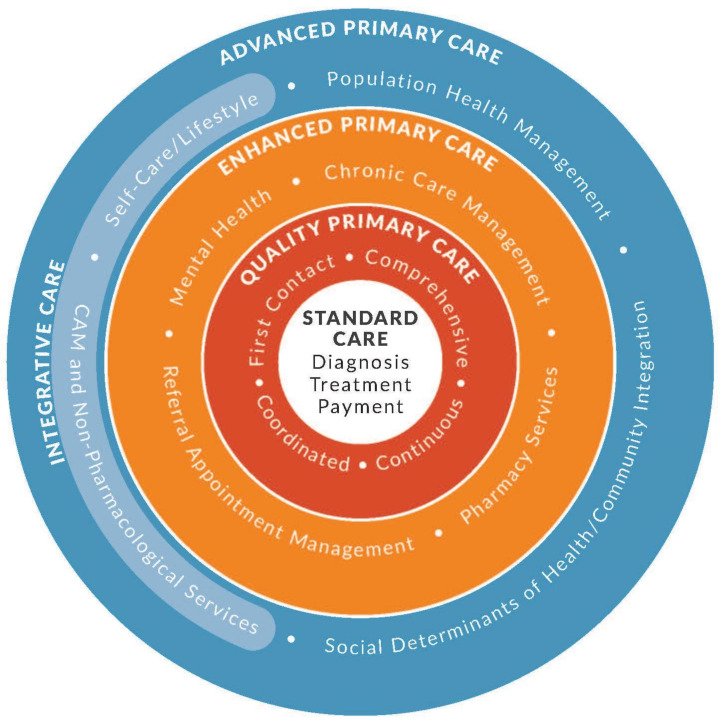
The Advanced Primary Care Model.

Integrative health care is part of a larger type of whole-person primary care we have recently described as Advanced Primary Care [16]. Briefly this model begins with the basic transaction with the patient, the encounter, and the therapy, but then surrounds that transaction with three additional circles of care that enhance or enable whole-person care. In the first circle are the components of the Starfield model of primary care that includes first contact, comprehensive and continuous care. Then, a second circle using enhanced management systems for care is provided to better integrate care delivery. This circle includes chronic disease management, enhanced care coordination, pharmacy services, and the integration of mental health. The fourth and final circle brings in support for lifestyle change and CAM and a process for addressing the social and economic determinants of health. Integrative health has a role in supporting behavioral and lifestyle determinants. Figure 5 illustrates this model. 

These models are only illustrative and not exhaustive of whole-person examples. To explore the impact that whole-person care models have on outcomes, we analyzed four aims of health care considered essential for quality. We used the outcomes described by the Institute for Healthcare Improvement called the Triple Aim [17,18] and added one additional aim (provider experience) to the analysis. The Triple Aim includes: (1) health improvement; (2) experiential outcomes in the form of patient satisfaction; and (3) cost reduction outcomes in the form of lowered costs per capita. 

## 4. Materials and Methods

We selected studies focused on the models of whole-person care and included a few that emerged from the search based on broader health system data. We included relevant examples of programs with elements of coordinated delivery in at least three elements: conventional medical care, complementary and alternative medicine, and self-care within the social context of the patient. We looked for outcomes based on the Triple Aim criteria, plus the provider experience. 

Two iterative Pubmed and Google searches were conducted between December 2020–May 2021 (last search done 15 May 2021) to seek studies looking at outcomes of whole-person outpatient primary care models. Search criteria used the search terms “Whole Health Veterans Administration”, “integrative medicine”, “integrative health”, “complementary and alternative medicine”, and, as they related to the outcomes, “health outcomes”, “cost-effectiveness”, “cost reduction”, “patient satisfaction”, “provider satisfaction”, and “physician satisfaction”. Inclusion criteria were models being in primary care, not done in a single sub-population or specially, not using a single modality or outcome measure, and only United States practices. After reviewing the citations from studies in the initial search, we conducted a second search, using the same criteria in Google. 

To explore the business impact more clearly, we then conducted a deeper dive into the Veteran’s Health System and Southcentral Foundation as existing models of whole-person care which have had extensive evaluation. We review and describe these findings in more detail. 

## 5. Results

The first search resulted in 1764 references. Eighty-three met the criteria for screening. Nine articles from this initial search were included in this review. Forty-two additional studies were then screened. Twelve of these sources were included for a total of twenty-one studies that met all criteria from which this narrative review follows. See Table S1 for a list of the studies reviewed. 

### 5.1. Evidence for Better Health Outcomes

The Veteran’s Administration (VA) health system implemented Whole Health as part of the Comprehensive Addiction and Recovery Act to reduce rates of opioid addiction and improve chronic pain management using complementary and integrative health approaches. Piloted at 18 sites, Whole Health had benefits on all major aims, according to a two-year evaluation of 133,476 veterans who began receiving Whole Health services in fiscal years 2018 and 2019 [19]. Whole Health is a relationship-focused “approach to healthcare that empowers and equips people to take charge of their health and well-being and live their life to the fullest” [20] and incorporates self-care and complementary and integrative health approaches [20,21]. 

Whole Health System (WHS) was evaluated using the self-reported Veterans Health and Life Survey, a standard survey used to measure outcomes across the VA. This study found that veterans with chronic pain who received WHS, compared to those who received usual care, reported engaging in more healthy behaviors, being more involved with health care decisions, made small improvements in purpose in life, wellbeing and quality of life. They also made improvements in their ability to manage chronic pain as measured by the Perceived Stress Scale [20]. This evaluation also assessed the impact of Whole Health opioids use and on overall pharmacy costs. WHS users with chronic pain had larger decreases in opioid doses than veterans who received usual care. Decreases were largest in veterans who used more WHS [20].

A study was done of the University of Arizona Integrative Health Center integrative medicine adult primary care clinic. That clinic model combines conventional and complementary medical treatments, including nutrition, mind–body medicine, acupuncture, manual medicine, health coaching, educational classes, and groups. Results from a real-world observational evaluation of patient-reported outcomes (*n* = 177) showed improvements in mental, physical, and overall health, work productivity, and overall well-being [22]. Specifically, those who participated spent less time at work impaired, had better sleep, less fatigue and pain, more consumption of vegetables and physical activity, and improvements in self-reported quality of life (SF-12 in general health items, physical component, and mental component) and improvements in depression symptoms, anxiety symptoms, and mental wellbeing (measured by WHO-5, PHQ-2 and GAD-2 scores) [22]. 

Health coaching is a non-reductionistic delivery method for whole-person care models. Health coaches use their expertise in human behavior to help individuals set and achieve health goals and are an increasingly important component of whole-person integrative care teams. In a case series of 5 lifestyle programs at a large Western PA integrated delivery system, 14,591 UPMC health plan members self-selected to participate in a health coaching program. There was no comparison group. Self-reported results at 180 days demonstrated that health coaching helped 77% of participants reduce stress, 50.5% of participants increased physical activity, 65.2% of participants improved nutrition, 44.2% of participants lost weight and 7% of participants quit tobacco [23]. Other studies of health coaching demonstrate improvements in medication adherence. A non-blinded, randomized control trial of health coaching (*n* = 224) versus usual care (*n* = 217) demonstrated an increase in self-reported medication adherence, 10% improved concordance of reported medications compared with documented medications in the medical record, and a 17% decrease in medications listed in the medical record that were not reported by the patient [24]. An Integrative Health coaching program for individuals with Type 2 Diabetes focused on individuals’ purpose in life and goals as a motivator for behavioral change. In a randomized control trial (*n* = 56), participants received 6 months of telephone coaching or usual care. Those receiving Integrative Health coaching had improved medication adherence, self- reported health, and improvements in hemoglobin A1c [25].

### 5.2. Evidence for Improved Patient Satisfaction

Regular users of integrative medicine like the “strong therapeutic relationship with a primary care provider who is a good listener and provides time, knowledge and understanding [26]”. They believe that an approach that uses CAM and conventional medicine together is better than either alone [26]. Veterans at VA sites piloting the Whole Health System of care reported higher ratings of patient-centered care for discussing care goals and less difficulties in care with their provider [20]. The University of Arizona Integrative Health Center study results demonstrated that all patients (100%) reported that their practitioners treated them with respect. On a 10-point scale (1 = worst and 10 = best), 89% of patients rated overall satisfaction between 7 and 10 and 93% of patients rated trust in their practitioner between 7 and 10. Almost all patients (97%) would recommend the program to others [27]. 

A study was done evaluating the University of Michigan’s Integrative Medicine Clinic. This clinic integrates conventional and complementary medicine using a whole-person model. A program evaluation of this clinic showed high patient satisfaction with the clinic. Patients were eligible for the study if they were seen at least 2 times in the clinic. Of the 274 patients initially surveyed, 85 completed the follow up questionnaire. More than half the patients rated their care as “excellent” or “best care ever” (37.6% and 24.7%, respectively). In resolving the primary issue, 55.3% of patients said the integrative medicine patient plan made “a significant difference” and 7.1% said that it “completely resolved my issue”. In rating the impact of visits to the clinic on overall quality of life, 82.4% of patients reported at least some improvement [28].

Multiple studies have been done on the model developed and delivered by Southcentral Foundation (sometimes called the Nuka system). This system uses a relationship-based, whole-person model care for Native Alaskan people. The Foundation’s approach includes calling and treating people as customer-owners rather than patients. This model has reported achieved excellent customer-owner satisfaction, with 96% satisfied with overall care and 95% who felt they had input into care decisions, and improved HEDIS measures [29]. These outcomes were a marked improvement over the more reductionistic, disease treatment approach taken for patients before the Nuka system was put in place. 

### 5.3. Evidence for Lower Cost

Although more research is needed, there are instances of all or parts of whole-person integrative care that indicate promising cost implications. VA Whole Health Care health care costs were lower for 133,476 veterans who received WHS compared to those who received usual care. Results showed 12% to 24% lower costs in all categories except drugs, and the calculated total cost savings was $4845 (20%) less per person using WHS [19]. Although medication costs increased across the board, the increase in drug costs was 4.1% lower for those who participated in WHS [19] than those not enrolled in Whole Health. Drug costs increased less for comprehensive WHS users than for veterans who received usual care. Comprehensive WHS users had at least eight visits including both core WHS and complementary and integrative health services. In veterans with mental health conditions (PTSD, anxiety and/or depression), annual increases in drug costs were 3.5% for comprehensive WHS users compared with 12.5% for veterans who received usual care. In veterans with chronic conditions, annual increases in drug costs were 5.3% for comprehensive WHS users compared with 15.8% for veterans who received usual care [20].

Other evidence for cost savings of coordinated conventional and complementary and alternative care comes from Alternative Medicine, Inc. (AMI), an integrative medicine independent provider association under contract with a large HMO in metropolitan Chicago. Doctors of Chiropractic served as primary care physicians specializing in non-surgical/non-pharmacological approaches and consulting with medical physicians as necessary. Analysis of clinical and cost outcomes based on claims and patient surveys of over 21,743 member-months for 4 years were compared with conventional medicine IPA performance for the same HMO product in same region and period, showed lower costs for integrative medicine patients compared to those who received usual care. Per 1000 patients, integrative medicine patients had 43% fewer hospital admissions, 58.4% fewer hospital days, 43.2% fewer outpatient surgeries and procedures and 51.8% lower drug costs [30]. Claims analysis after 7 years demonstrated that compared to conventional medicine IPA performance, AMI’s members had 60.2% fewer admissions, 59% fewer hospital days, 62% fewer outpatient surgeries and 85% lower pharmaceutical costs [31].

A study was done of the Maharishi Vedic (MV) approach, another multicomponent prevention program that integrates meditation, yoga, nutrition recommendations, supplements and breathing exercises. Researchers conducted a retrospective study using Blue Cross/Blue Shield Iowa data for patients who received care under the MV approach (*n* = 693), compared the statewide norm (*n* = 600,000) and a control group (*n* = 4148) getting non-holistic care. Four-year total medical expenditures for the MV approach group were 59% lower compared to statewide norm and 57% lower than the control group. Total medical expenses were 63% lower than the statewide norm at 11 years. Across ages and disease states health care utilization was lower for patients who received care under the MV approach [32]. 

We found several other program evaluations and randomized studies combining self-care with non-pharmacologic services and conventional care for individuals with chronic disease with most showing lower costs. We do not review all of those here but mention some of the major ones for illustration. The Multicenter Lifestyle Demonstration Project reported that for persons with coronary artery disease, lifestyle changes plus yoga, meditation and progressive relaxation was medically effective and safe compared with revascularization surgery. Average cost savings of $29,529 was calculated for those who participated in the lifestyle change group [33]. The multicenter Diabetes Prevention Program for patients with prediabetes showed that lifestyle changes delayed development of diabetes and reduced incidence more than the drug metformin. Those in the lifestyle group had an 11-year delay and 20% lower total incidence of developing diabetes compared to a 3-year delay and 8% lower total incidence for those taking metformin compared to conventional care alone. The cost of lifestyle changes to society was about $8800 per quality-adjusted life-year (QALY) saved compared to about $29,900 for metformin [34]. 

Integration and coordination are essential to whole-person integrative care. Collaboration between health care and social services can improve health outcomes while decreasing costs. An example of a closed loop referral program is WellCare’s that provides referrals to community-based public assistance programs, such as housing services and utility assistance, for 2718 participants insured through Medicare Advantage or Medicaid managed care in 14 states. Medical cost savings in the year after the needs were met, compared to those who had no needs met, were $1500 for participants with at least 1 social need met and about $2443 (10% lower) for participants with all social needs met [35]. 

Health coaching is an evidence-based process that assists patients in engaging and empowering them for their own health improvement through behavior change. It may reduce cost while improving health outcomes, independent of the specific treatment modality. Administrative claims data for high-cost commercial health plan members with multiple comorbidities and/or high adjusted clinical group risk scores were compared for members who received health coaching (*n* = 1161) and members who did not receive health coaching. Health coaching led to significant reductions in outpatient and total expenditures with estimated outpatient savings of $286 per person per month and estimated total cost savings of $412 per person per month [36]. 

### 5.4. Evidence for Improved Clinician Experience

Physicians who practice all or part of whole-person integrative care are less likely to burn out than other physicians. VA Whole Health Care evaluation demonstrated that clinical staff who are more involved with Whole Health were less likely to resign. All employees who were involved with Whole Health were more engaged and less likely to experience burnout. Employees reporting any involvement in Whole Health in 2019 identified their VA as a Best Place to Work and reported better leadership, intrinsic motivation, and being more engaged due to the behaviors of their supervisors [20].

In another example, physicians who perceived that their clinic was able to meet patients’ social needs were less likely to be burned out according to an analysis of 1298 family physicians in ambulatory primary care settings [37]. Employee turnover at Southcentral Foundation, which provides relationship-based, whole-person care for Native Alaskan people, decreased by 15% from 2007 to 2015 [29]. 

Clinicians trained in complementary and alternative approaches appear to have improved satisfaction and wellbeing when integrated into usual care. Acupuncture training was significantly associated with decreased depersonalization of patients, a factor in burnout, according to a survey of 233 family physicians at the Uniformed Services Academy of Family Physicians 2017 conference [38]. Foundations in Integrative Health piloted a 32-h online competency-based interprofessional course in integrative health and evaluated results for 214 providers who completed the course. These providers had statistically significant improvements in their resiliency, changes in several wellness behaviors such as days engaged in an activity to manage stress, personal mind–body spiritual practices (breathing, spiritual rituals, and personal reflection), moderate, 30-min daily exercise, socializing with friends, and doing hobbies [39].

## 6. Discussion

In this review we have provided definitions and descriptions for several models and frameworks used to provide whole-person care rather than more reductionistic treatment models that address specific disease treatments only. In addition, we have explored the evidence of the impact of these models on four main types of outcomes—clinical, patient satisfaction, provider burn out and cost. 

While most of these models demonstrated positive benefit in all these outcome areas, they are quite heterogenous and unfocused (See Table S1). There is no unified theory for whole-person care. Dimensions of what constitutes a whole person varied in different models, and they often had differing emphasis or strategic steps in their application, some using diet as major focus while others using mind–body approaches, for example. More work needs to be done to synthesize and simplify these models into more coherent systems. This might be done by studying the underlying mechanisms of healing, independent of looking at specific treatments for specific outcomes as is done in reductionism. Two lines of research may help improve this situation.

One research strategy for better understanding whole-person care is to look at so-called placebo response processes. Placebo effects cut across systems of care as they illuminate underlying mechanisms of healing that are agnostic to the specific treatments used. Mechanisms for placebo responses involve belief and expectation, classical conditioning, and social learning as they are embodied in the ritual of therapy [40]. The release of underlying healing processes by these mechanisms occurs across multiple conditions, irrespective of the modality or treatment being used. Thus, it is non disease specific and non-reductionistic. This research area may allow for are more complete understanding of whole-person healing and the integration of ancient traditional, complementary, conventional, allopathic, homeopathic or integrative systems, all of which utilize the placebo response. By studying the mechanisms of healing words and rituals, we may get to the core aspects of collective and individual healing across all health care systems [41].

A second area for research that could shed light on these fundamental processes of healing would be on non-instrumental approaches to healing. These go by a number of names such as intention, the biofield, subtle energies or consciousness and use ancient techniques such as laying on of hands, spiritual healing and more modern approaches such as Therapeutic or Healing Touch and Reiki [42,43]. This also includes more conventional approaches to healing such as psychotherapy, mind–body practices, and hypnosis in various forms. These are often collectively referred to as “vitalist” approaches. Researching these non-instrumental approaches by examining “healing presence” and communication processes in the patient-provider encounter could shed light on fundamental processes of whole-person healing and how they work independent of the specific interventions that use a drug, pill, needle or knife linked to a specific outcome. [44] More research on the impact of these health models is needed. 

## 7. Conclusions

Current approaches to health care rest on reductionistic research strategies that seek treatments found by isolating and studying parts of a human being and then applying them for their “specific effects”. This is the basis of so-called evidence-based medicine. However, people are not just a collection of their parts. They are complex, dynamic, interactive systems that need to be studied and treated using whole person, ecological models. This review explored several examples of such holistic models and found improved outcomes from their use on patient satisfaction, health outcomes, and on reduced costs and reduced provider burnout. While there is increasing evidence for the benefit of these models, there is a need for more and better research using whole system strategies. We need to explore and standardize our understanding of healing and use both research and delivery strategies more aligned with the nature of actual human beings.

## Supplementary Materials

The following are available online at https://www.mdpi.com/article/10.3390/medicina57070677/s1, Table S1: Studies included in narrative review.
