# Supplementary material for: The Case for Whole-Person Integrative Care
Source: Medicina (Kaunas). 2021 Jun 30;57(7):677. doi: 10.3390/medicina57070677 (PMC8307064; doi:10.3390/medicina57070677)
Supplement: Supplementary file 1 [file medicina-57-00677-s001.zip › medicina-1244996-supplementary.pdf]

Table S1: Studies included in narrative review

	Study	Model type	Program	Type of Study	Aim	Outcome measure
1	Bokhour, B., et al (2020).	Whole Health	VA System	Mixed method evaluation	Better Health, Lower cost, Improved Provider experience, Improved patient satisfaction	Improvements in self-reported wellbeing and quality of life, perceived stress, ability to manage chronic pain, decreased use of opioids, higher ratings of patient centered care, improved Whole Health employeessatisfaction with less burnout, less likely to resign
2	Brooks, A.J., et al (2020).	Integrative Health Training	Foundations in Integrative Health	Survey of participants completing 32 hour course	Improved Provider experience	Improved resilience, wellness behaviors
3	Budzowski, A.R., et al (2019).	Health Coaching	UPMC health plan	Case series 5 lifestyle programs health plan members, no comparison group	Health outcomes	Reduced stress, increase physical activity, improvements in nutrition, increased weight loss
4	Crawford, P.F, et al (2019).	Evidence based Non-pharmacologic intervention (Acupuncture) training	Attendees of Uniformed Services Academy of Family Physicians	Conference survey of all participants	Improved Provider experience	Reduced depersonalization of patients for those with acupuncture training

5	Crocker, R.L., et al (2017).	Whole Person Primary Care	University of Arizona	Fidelity study, random sampling of clinic patients	Improved patient experience	Patient satisfaction, high empathy scores, patient satisfaction and likelihood to recommend program to others
6	Crocker, R.L., et al (2019).	Whole Person Primary Care	University of Arizona	Real world observation study, pre- and post-survey	Better Health	Improvements in health related quality of life, work productivity, overall wellbeing, mental health measures, improved sleep, improved nutrition, improved physical activity, decreased pain
7	De Marchis, E., et al (2019).	Social care program	Ambulatory primary care applicants for ABFM continued certification	Cross-sectional survey analysis	Improved Provider experience	Less burnout
8	Eby, D. (2016).	Relationship based care	Southcentral Foundation	QI analysis	Better health, Improved Patient Satisfaction, Improved Provider experience	Improved HEDIS measures, improved satisfaction with overall care and shared decision making, decreased employee turnover

9	Gaudet, T. and Kligler, B (2019).	Whole Health	VA health system	Commentary		
10	Herman, W.H., et al (2005)	Lifestyle program	National Diabetes Prevention Program	Cost-effectiveness analysis	Better Health, Lower costs	Delay and reduced incidence of diabetes cost effectiveness
11	Jonk, Y., et al (2015).	Health coaching		Case controlled claims data analysis	Lower cost	Reduced outpatient and total medical expenditures
12	Leinenkugel, J., et al (2020).	Whole Health	VA System	VA System medical cost data before and after implementation of Whole Health	Reduced Cost	Decreased cost of total care, decrease pharmaceutical costs, Recommendation to transform VA healthcare delivery to person-centered, relationship based, recovery focused care
13	McCaffrey, A.M., et al (2007).	Whole person Primary Care	Marino Center, Cambridge, MA	Focus group	Improved Patient Satisfaction	Satisfaction with IM providers, Dissatisfaction with conventional medicine
14	Myklebust, M., et al (2008).	Whole Person Primary Care	University of Michigan Integrative Medicine Clinic	Prospective patient survey study	Improved Patient satisfaction, Better Health	Improved holistic health quality, improved SF-12 scores and improved patient satisfaction

15	Orme-Johnson, D.W. and Herron, R.E (1997).	Multicomponent program		Retrospective study with health plan claims	Lower costs	Reduced total medical expenditures, lower hospital utilization
16	Ornish, D (1998).	Multicomponent program		Multicenter study lifestyle versus control revasculariza tion surgery	Better Health, Lower cost	Cost savings Reduced Angina
17	Pruitt, Z., et al (2018).	Social care program	WellCare	Claims data analysis	Lower cost	Reduced medical cost
18	Sarnat, R.L. and Winterstein, J. (2004).	Whole Person Primary Care	Integrative Medicine IPA in Chicago, IL (PCP are Doctors of Chiropractic)	Analysis of clinical and cost outcomes using claims data	Lower costs	Fewer hospital admission, hospital stays, outpatient costs and pharmaceutic al costs
19	Sarnat RL, and Winterstein J. (2007).	Whole Person Primary Care	Integrative Medicine IPA in Chicago, IL (PCP are Doctors of Chiropractic, Integrative M.D. and D.O. physicians)	Analysis of clinical and cost outcomes using claims data	Lower costs	Fewer hospital admission, hospital stays, outpatient costs and pharmaceutic al costs
20	Thom, D.H., et al (2015).	Health Coaching	"Safety-net" health center in San Francisco	Non-blinded RCT health coaching versus usual care for patients with diabetes, hypertension or hyperlipidem ia	Better Health	Improved medication adherence
21	Wolever, R.Q., et al (2010).	Health Coaching	Duke University Integrative Medicine	RCT telephone health coaching or usual care	Better health	Improved medication adherence, Improved self-reported

						health, and improvements in A1c
